# Behavioral effects of environmental enrichment on male and female wistar rats with early life stress experiences

**DOI:** 10.3389/fphys.2022.837661

**Published:** 2022-09-26

**Authors:** K. Corredor, J.M. Duran, L. Herrera-Isaza, S. Forero, J.P. Quintanilla, A. Gomez, G. S. Martínez, F. P. Cardenas

**Affiliations:** ^1^ Laboratory of Neuroscience and Behavior, Universidad de los Andes, Bogotá, Colombia; ^2^ Centro de Investigación en Biomodelos, Bogotá, Colombia

**Keywords:** environmental enrichment, adverse childhood experiences, anxiety, cognition, social interaction, sex, behavior, rats

## Abstract

Exposure to adverse childhood experiences or early life stress experiences (ELSs) increase the risk of non-adaptive behaviors and psychopathology in adulthood. Environmental enrichment (EE) has been proposed to minimize these effects. The vast number of methodological variations in animal studies underscores the lack of systematicity in the studies and the need for a detailed understanding of how enrichment interacts with other variables. Here we evaluate the effects of environmental enrichment in male and female Wistar rats exposed to adverse early life experiences (prenatal, postnatal, and combined) on emotional (elevated plus maze), social (social interaction chamber), memory (Morris water maze) and flexibility tasks. Our results—collected from PND 51 to 64—confirmed: 1) the positive effect of environmental enrichment (PND 28–49) on anxiety-like behaviors in animals submitted to ELSs. These effects depended on type of experience and type of enrichment: foraging enrichment reduced anxiety-like behaviors in animals with prenatal and postnatal stress but increased them in animals without ELSs. This effect was sex-dependent: females showed lower anxiety compared to males. Our data also indicated that females exposed to prenatal and postnatal stress had lower anxious responses than males in the same conditions; 2) no differences were found for social interactions; 3) concerning memory, there was a significant interaction between the three factors: A significant interaction for males with prenatal stress was observed for foraging enrichment, while physical enrichment was positive for males with postnatal stress; d) regarding cognitive flexibility, a positive effect of EE was found in animals exposed to adverse ELSs: animals with combined stress and exposed to physical enrichment showed a higher index of cognitive flexibility than those not exposed to enrichment. Yet, within animals with no EE, those exposed to combined stress showed lower flexibility than those exposed to both prenatal stress and no stress. On the other hand, animals with prenatal stress and exposed to foraging-type enrichment showed lower cognitive flexibility than those with no EE. The prenatal stress-inducing conditions used here 5) did not induced fetal or maternal problems and 6) did not induced changes in the volume of the dentate gyrus of the hippocampus.

## 1 Introduction

### 1.1 Early life stress

The National Survey of Child and Adolescent Well-Being II revealed that over a third of the sample have experienced three or more adverse situations during childhood in the United States (Garcia et al., 2017). It has also been estimated that poverty levels in children and adolescents in Colombia reached 44% before the pandemic, and rised to 53% during the crisis in 2020 (Núñez, 2021), which results in a lack of the necessary resources to fulfill physical, emotional and social needs. This deprivation of key experiences or material means during key periods of the development is known as *Adverse Childhood Experiences (ACEs)* in a human context (Gilgoff et al., 2020) or Early Life Stress (ELS) experiences, in a more general animal model context. ELSs generate negative effects on physiological, psychological, and social functions, and increase vulnerability to physical and psychological pathologies (Anda et al., 2006; Krugers et al., 2017). Experiences leading to ELS include physical, psychological, and sexual abuse, neglect, chronic illness in close relatives, intrafamily or environmental violence, and parental drug addiction (Finkelhor et al., 2013; Bethell et al., 2014). One of the more severe effects of ELSs is the perception of loss of control, that has been related to the development of stress and helplessness (Overmier and Seligman, 1967; Drugan et al., 1997).

ELSs have consequences in all areas of individual adjustment (Sánchez et al., 2001; Lovallo et al., 2013; Salinas-Miranda et al., 2015; Chen et al., 2016; Filipkowski et al., 2016; Krugers et al., 2017) and show effects at the social and personal level of functioning (Iverson et al., 2013; Bellis et al., 2014). ELSs are also related to development of psychopathologies such as anxiety disorders, depression, and substance abuse (Heim and Nemeroff, 2001; Nemeroff, 2004; Fumagalli et al., 2007; Moffett et al., 2007; Feldman, 2015; McLaughlin, 2016) during adulthood (Martínez, 2008). It has been reported that severe ELSs affect neuronal structure and function, as well as the morphology of structures that are responsible for emotional, social, and cognitive responses, such as the hippocampus, amygdala, ventromedial prefrontal cortex, and developing cortical-limbic pathways (Gorka et al., 2014; McLaughlin et al., 2014; Villegas et al., 2015). Even with a significant amount of research using animal models to elucidate the effects of ELSs, few works have focused on the effect of pre-and postnatal stressors (Gómez-González and Escobar, 2009), and their cumulative effect over different behavioral areas (Cannizzaro et al., 2006).

### 1.2 Environmental enrichment

The use of animal models has enabled to establish that many environmental enrichment approaches could be used to reduce, prevent, or mitigate the effect of ELSs. Environmental enrichment (EE) refers to all the physical and social changes made to the animal environment. These environmental changes allow better performance of the individuals in different domains, in comparison with animals in standard environments (Redolat and Mesa-Gresa, 2011; Hannan, 2014; Mora-Gallegos et al., 2015). These changes can also revert some of the detrimental effects of ELSs and even improve the cognitive dysfunctions caused by them (Francis et al., 2002; Nithianantharajah and Hannan, 2006; Pamplona et al., 2009). EE has also been shown to improve the development of social (Kambali et al., 2019), cognitive, emotional (Pamplona et al., 2009; do Prado et al., 2016), and memory skills (Aronoff et al., 2016), and also influence processes such as brain plasticity (Barros et al., 2019; Ohline and Abraham, 2019), neurological pathologies (Livingston-Thomas et al., 2016) and disorders such as autism spectrum (Woo et al., 2015). However, efforts to create a systematic characterization of how various categories of environmental enrichment interact with behavior have not succeeded and therefore, an immense variability of protocols with widely varying results exists.

Enrichment protocols vary in features such as *intensity* (Grimm and Sauter, 2020) and *duration* (Bhagya et al., 2017), *age of exposure* of the subjects (Yang et al., 2007; Baldini et al., 2013; Vivinetto et al., 2013), *types of controls* used in the experiments (Hannan, 2014), the *goals pursued* by its implementation (Li and Tang, 2005; Solinas et al., 2010; Hannan, 2014) and, in general, how EE is defined by the researchers (Connors et al., 2015). A second source of variability is the experimental *moment* (before, during or after the administration of other variables) in which EE is applied in the experimental design (Green et al., 2006; Milgram et al., 2006; Redolat and Mesa-Gresa, 2011). The third source of variability is the *assessment of the effect* of EE on different types of responses, which varies from one research to another. For example, anxiety can be tested in the elevated plus maze, the open field or in the defensive burying test (Paez-Martinez et al., 2013; Ahmadalipour et al., 2015; Gong et al., 2018); memory in the Morris water maze (Vorhees and Williams, 2006; Paul et al., 2007) or in the object (or place) recognition test (Tata et al., 2015), among others. This may generate differential effects of EE in the tested domains, depending on the assessment strategy. The last source of variability is the *characteristics of the animals used* in the experiment; in fact, species (Baumans, 2005; Emack and Matthews, 2011), strain (Tsai et al., 2002), sex (Skwara et al., 2012; Girbovan and Plamondon, 2013), and genotype (Wood et al., 2010) interact differentially with the environmental variable.

All these sources of variability make it difficult to understand the precise mechanisms of EE behavioral effects (Solinas et al., 2010; Woo et al., 2015) and also an become an obstacle to specify the EE therapeutic potential. For this reason, it is important to identify the mechanisms responsible for the effects of EE to provide clear parameters for the EE protocol implementation.

### 1.3 Sex differences

It is also important to note that in despite of the well documented sex differences in prevalence, and etiology of the pathologies, as well as response to treatment in both humans (Palanza, 2001; Thibaut, 2016; Pinares-Garcia et al., 2018; Green et al., 2019) and animals (Anker and Carroll, 2011; Keeley et al., 2014, 2015; Becker and Koob, 2016), it is customary for traditional biomedical research to systematically omit the use of females in animal experimentation (Will et al., 2017). Considering sex as a relevant variable in animal models can help explain contradictory findings, such as the differential effect of drugs (Kokras et al., 2015), and avoid delays in expanding knowledge (Cahill, 2006; Miller et al., 2017). Having a better understanding of sex differences, will enable to develop more accurate experiments and research protocols.

Specifically, the effect of ELSs on males and females has been document to be different. For instance, fetal exposure to alcohol generates a greater increase in the fear response of females compared to males (Osborn et al., 1998). On the other hand, after being exposed to maternal separation during the breeding period, females show the highest activity level of Cytochrome C oxidase, an enzyme involved in the metabolism of energy sources in the brain (Spivey et al., 2011). The surge of this enzyme in turn increases metabolism in cells that promote cellular oxidative stress, which results in increased neuronal death (Zuluaga Vélez and Gaviria Arias, 2012).

In the case of EE as an intervention strategy, there is also evidence of differential effects due to sex (Girbovan and Plamondon, 2013). Females may be more sensitive to the anxiolytic effect of EE in behavioral paradigms such as elevated plus maze and forced swim test (Skwara et al., 2012; Hendershott et al., 2016). Currently, it is difficult to find research comparing the differential effect of EE, or other variables, using sex as a biological variable.

For a long time, it was assumed that the use of females in behavioral and neurophysiological research implied an increase in variability, and, therefore, creates an inconsistency in the results, due to cyclic hormonal changes specific to their sex. In 2019 Scholl et al. reported that there are no estrous-cycle-related differences in anxiety tests, such as the elevated plus maze. In that study, no differences were neither found in the social interaction test that could be associated with the estrous cycle (Scholl et al., 2019). Based on this evidence it is plausible to assume that it is possible to use groups of females without having to separate them according to the phase of their estrous cycle, at least in above mentioned tests.

This work aims to the analyze of the effect of two EE strategies (physical and foraging) on behavioral tests (emotion, memory, and cognition) and neuroanatomical features (dentate gyrus of the dorsal hippocampus), in both male and female Wistar rats exposed to three types of ELSs (prenatal, postnatal, and combined stress). In this work, it is expected that the use of prenatal and postnatal stress generating stimuli, of lower intensity than those traditionally used in research, will lead to less profound alterations, which can be corrected using a brief protocol of environmental enrichment. In this situation, there is a risk of finding no marked effects of the stressful stimuli, but the experimental situation would more closely resemble the human conditions of early “maltreatment” experiences, such as the absence, for prolonged periods, of contact with the mother (as in the case of working mothers who must leave their children for many hours a day). It is possible that some changes in emotional processing, as well as cognition, may be attributed to these types of stressors and also reversed by enrichment. It is even hypothesized that changes in social interaction processes may be evidenced.

This study provides further knowledge about how early life experiences can differentially affect males and females. In the same direction, this work also shades light on how different categories of environmental enrichment affect behavior, and how its effects depend on the sex of the individual. It is the aim of the authors that our data will contribute to the improvement of the understanding on the relationship of these variables and will motivate future work and the development of applications for environment enrichment.

## 2 Materials and methods

### 2.1 Animals

48 female and 24 male Wistar rats were obtained from the National Institute of Health of Colombia to start the breeding of control and experimental animals at the beginning of the study. All animals were housed in groups of five individuals of the same sex using polycarbonate cages (16.5 cm × 50 cm × 35 cm). Cages were kept under controlled environment with a 12 h light/12 h dark cycle (lights on at 06:00), *ad libitum* access to food and water, constant room temperature of 22 ± 2°C, and humidity of 57 ± 10%. During the mating period of 10 days, cages housed one male and two virgin females. Females were checked daily for the appearance of a vaginal plug and body weight was recorded. Once pregnancy was confirmed by the appearance of vaginal plug, females were housed individually. Gestational days (GD) began to be counted from this point as GD 1 to GD 21.

The number of neonates that died before weaning (PND 21), in the prenatal, postnatal, and combined stress groups, was eight animals. This is equivalent to 4% of the population, which is a value similar to that expected under normal rearing conditions in our laboratory.

All procedures were conducted according ethical and legal standards required for research using laboratory animals in Colombia (Law 84 of 1989 and Resolution No. 8430 of 1993 of the Ministry of Health) and the research project was approved by the Ethics Committee for the use and care of Laboratory Animal of the University of Los Andes—CICUAL Uniandes (C.FUA_17–015).

### 2.2 Early life stress experiences

Four groups were designed to reflect the early life stress experiences variables. The control group used 29 males and 32 females, and three active stress groups were created as follows.

#### 2.2.1 Prenatal stress

**FIGURE 1:**
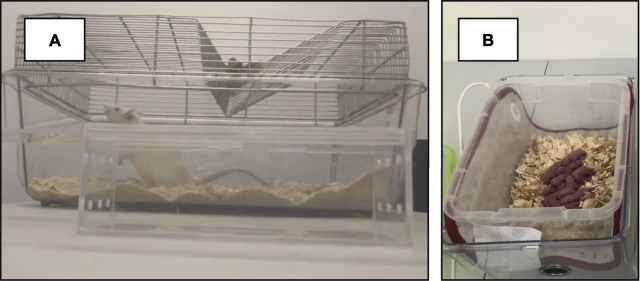
Early Life Stress. **(A)** Restrainer container for prenatal restraint stress. **(B)** Isolation box for pups during maternal separation stress. After the pups removal from the housing cage, the dam was returned to the housing room.

The protocol followed here is the same as reported by several authors, including Baier et al. (2012) and Li, et al. (2014). Once the pregnancy status of the females was confirmed, the gestational monitoring procedure was initiated. At the beginning of each stress essay, the dam was moved from the housing room to an adjacent experimental room and placed in a clear plastic restrictor (18 cm × 6 cm × 7 cm). In this restriction box, the female had sufficient breathing space (Figure 1A). This procedure was performed 3 times a day for 45 min. The restriction periods were randomized in three schedules (8:00–11:00; 11:00–14:00 and 14:00–16:00) to avoid habituation. In order to avoid habituation, each of the three 45-min restriction periods for each female could be between 8:00–11:00, 11:00–14:00 and 14:00–16:00, and was changed every day, avoiding that the end of one of the restriction periods would match the beginning of the next one. The restriction criteria were selected to guarantee the maintenance of the welfare conditions for both mothers and offspring, as recommended by Baier et al. Restraint sessions were performed between GD 12 and 18 (Baier et al., 2012; Li et al., 2014). 31 males and 28 females were submitted to this type of stress.

#### 2.2.2 Postnatal stress

The day of birth of the pups was counted as postnatal day one (PND 1) and no manipulation was performed. On PND 2 to 14, the pups were removed from the nest for 180 min daily, weighed and placed in an isolation box in an adjacent experimental room (08:00 to 11:00). Pups from the same litter were placed together inside an isolation box (Figure 1B). At the end of the separation period, pups were returned to their home cage. The maternal separation protocol has been previously validated and described by Couto et al. (2012). The temperature of the isolation box was maintained at 32°C using thermal blankets. On PND 21, the offspring was weaned. This condition included 32 males and 26 females.

#### 2.2.3 Combined stress

Animals assigned to this condition were exposed to both prenatal and postnatal stress protocols as described above. For this condition 26 males and 29 females were used.

### 2.3 Environmental enrichment

At PND 22, litters were divided by sex and housed in standard laboratory conditions in groups of 5 same-sex animals, for a 7-days acclimation period. Subsequently, animals were randomly assigned to one of three environmental enrichment conditions: 1) physical (passive) enrichment (n = 41 males and 41 females); 2) foraging (active) enrichment (n = 38 males and 37 females); or 3) standard-control housing (non-EE; n = 39 and 37 females). The environmental enrichment elements were introduced into the standard home cages accordingly to the enrichment condition from PND 28 to 49.

#### 2.3.1 Physical enrichment

**FIGURE 2:**
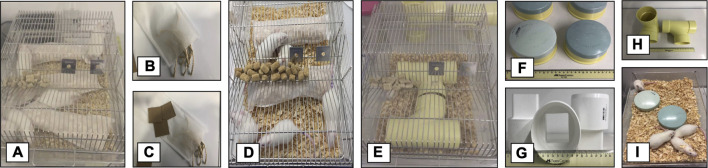
Environmental enrichment. **(A,D)** Foraging-environmental enrichment disposition at the home cage. **(B,C)** Material used for foraging-environmental enrichment. **(E,I)** Physical-environmental enrichment disposition at the home cage. **(F,G,H)** Material used for physical-environmental enrichment.

The use of physical enrichment is not a systematic practice. There are many protocols using very different objects (Pinelli et al., 2017). Here we use three different shapes of PVC pipe to modify the physical features of the home cage, providing access to new surfaces and hiding places, allowing the interaction with novel objects. To prevent the familiarization with the objects, they were changed every 3 days (Figures 2E–I). The PVC objects were placed inside the housing box. This kind of stimuli does not imply an active interaction with the animal and for this reason we considered it as passive enrichment.

#### 2.3.2 Foraging enrichment

There are very few reports in the literature dealing with foraging as a strategy for environmental enrichment. Hobbiesiefken et al., used foraging material as enrichment for mice (Hobbiesiefken et al., 2021). However, in their study the material was delivered in a direct manner (unpacked), minimizing the requirement for an active process to get it. Previous works in our laboratory (unpublished data) showed that the use of packed bedding material increases the activity of the animals. Thus, for this type of foraging enrichment, three bags of teabag paper (30 × 30 cm) filled with bedding material, cardboard pieces (5 × 5 cm) or organic rope pieces (10 cm), were placed in the home cage. Every third day the packed material was changed (Figures 2A–D). In this condition, the animal has to engage in an active interaction with the stimuli; for this reason, we considered foraging as an active enricher.

### 2.4 Behavioral testing

All experiments were recorded and digitized for analysis using the software Any-Maze (Stoelting Co.). The software X-plo-Rat 3.3 was also used to record specific behaviors.

#### 2.4.1 Detailed procedure

**FIGURE 3:**
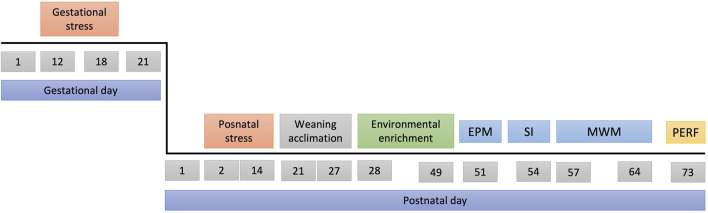
Experimental design. Top line: gestational days, bottom line: postnatal days. Abbreviations EPM, Elevated Plus Maze; SI, Social Interaction; MWM, Morris Water Maze; PERF, trans-cardiac perfusion.

The breeding of the experimental animals began with 48 female and 24 male Wistar rats. Each cage housed one male and two virgin females for 10 days, with a 12 h light/12 h dark cycle (lights on at 06:00). Once pregnancy was confirmed (GD1), females were individually housed. A total of 233 Wistar rats (115 female and 118 male) were used as experimental subjects. The timeline of the experiment is presented in Figure 3. Some dams were randomly selected to receive the prenatal stress protocol between GD 12 and GD 18 (for details on this protocol refer to section “2.2.1. Prenatal stress”). After delivery (GD 21), litters were culled to ∼8–10 pups per cage, and postnatal days (PND) were calculated from the date of birth. Pups were then randomly selected to receive postnatal, combined or no postnatal stress. For details on postnatal stress inducing protocols, please refer to section “2.2.2. Postnatal stress”. On PND 22 litters were regrouped by sex and housed in cages containing up to 5 animals. From PND 28 to PND 49 rats were randomly assigned to one of three environmental enrichment conditions: Physical enrichment (for details on the protocol please refer to section “2.3.1. Physical enrichment”); Foraging enrichment (for details on the protocol please refer to section “2.3.2. Foraging enrichment”); or no environmental enrichment or standard condition.

On PND 51, the collection of behavioral data begun, as follows.

#### 2.4.2 Elevated plus maze

To assess anxiety-like behavior, rats were tested on an elevated plus maze (EPM). Subjects were tested only once on this apparatus at PND 51. Briefly, the elevated plus maze consists of two open (unprotected) platforms (open arms) crossed at 90 degree-angles by two platforms enclosed by 40 cm high walls (closed arms). Each arm is 50 cm long × 10 cm wide. To prevent rats from falling, a rim of plexiglass (1 cm high) serves as a wall. The apparatus is made of wood covered with black melamine and elevated 50 cm above the floor. The structure is placed in an isolated test room, lit by white LEDs (60 Lux in the center of the maze). Each rat was tested only once and had never been exposed to the apparatus before. The test always began by placing a rat in the center of the maze with its nose facing one of the closed arms and allowing it to explore the maze for 5 min. Once the test was completed, the animal was returned to the home cage, and the maze was thoroughly cleaned, using 10% alcohol and disposable paper towels. The experiment was recorded and digitalized for further analysis. Both, the number of entries and the amount of time spent in each type of arm were recorded. An entrance into an arm was defined as the placement of all four paws in the surface of the arm. A full day of recovery before the next behavioral experiment was allowed to each animal.

#### 2.4.3 Social interaction test

Crawley’s social interaction test (SI) is based on the subject’s free choice to explore the environment (Crawley, 2004). The SI allows assessing the time each subject spends exploring the arena and the time spent in social approach behaviors, generating a permanence index. The test was conducted in a three-chambered Plexiglas box (31 × 43 × 30 cm in each compartment) and two movable barriers (43 × 0.05 × 30 cm) between the compartments.

On PND 53 each rat was placed in the center chamber for a 5-min habituation period. After these 5 min the barriers were removed allowing the animal to explore the two compartments that contained a wire box. After these 5 min, two wire cages were placed at opposite ends of the arena. One contained an unfamiliar animal while the other was empty. The wire cage allows visual, olfactory, and auditory interaction during the test period. The time spent in each chamber of the arena, as well as the interactive behavior with both cages, was recorded.

Twenty-four hours after the first trial (PND 54), the same procedure was conducted but this time a second new animal was introduced into the previously empty wire cage. During the session, the time devoted to explore the arena and the time the subject interacted with each of the two peers inside the wire cages was recorded (Crawley, 2004). Two full days of recovery before the next behavioral experiment was allowed for each animal.

#### 2.4.4 Morris Water Maze

Subjects were habituated, trained, and evaluated in a spatial memory paradigm including a cognitive flexibility task on the Morris water maze (MWM). The test was conducted in a round black pool (200 cm diameter and 90 cm deep) filled to a depth of 75 cm with warm water (at 22–26°C). The pool included visual cues that indicated north, south, east, and west points from the center, and the apparatus was divided into four quadrants. The escape platform was a 10 cm diameter × 70 cm height Plexiglas tube, placed in the center of one quadrant and submerged 2 cm beneath the water surface.

The animals were trained to find the hidden platform in daily sessions of 4 trials for the first day (PND 57), and 10 trials for the next 6 days. For the test (PND 63), the platform was removed from the pool, and the animal was allowed to swim freely for 120 s in a single trial. The time spent in each of the quadrants was recorded.

Twenty-two hours later (PND 64), the cognitive flexibility assessment was conducted. The escape platform was placed in a different quadrant and the latency to find the new platform location was recorded. A flexibility index was calculated as the magnitude of the difference between the latencies from training and reversal sessions. This index serves as an indicator of how fast the animal was able to acquire the new rule (new platform location).

### 2.5 Neuroanatomical changes

#### 2.5.1 Brain histology

At PND 73, subjects were perfused trans-cardially with saline solution (0.9%), followed by a 4% paraformaldehyde solution to fix the brain for collection. Six days after the collection one of the two hemispheres was cut in coronal sections (15um) with a Compresstome^®^ VF-300. Up to sixteen slices for each animal were analyzed from a sample of four to six subjects for each experimental group. For this, every 20th section was collected using the systematic uniform random sampling (ssf = 1/20).

The volume of the dentate gyrus was estimated using the Cavalieri equation: V=∑n[T(ssf)(a)] ; where *n* is the number of observations; T is the average thickness of every slice sampled (15um); *ssf* is the sampling fraction (1/20) and a is the area for each observation (Sadeghinezhad and Amrein, 2021). Sections were mounted, dried, and stained with cresyl violet. Here we report data obtained for the dentate gyrus of the dorsal hippocampus; an area related to emotional reactions (Ballesteros et al., 2014; Postel et al., 2021). Images of the area of interest were taken with an optic microscope (MEIJI MT4200H) and a digital camera Canon (EOS—850D) of 20Mp. The slices were analyzed using the software ImageJ 1.8.0_172. The areas of interest were delimited using the rat brain atlas (Paxinos and Watson, 2006).

### 2.6 Statistical analysis

The software R for statistical analysis (R Development Core Team, 2020) was used to eliminate atypical data. Subsequently, missing data were imputed for each variable using linear regression. Normality (Shapiro-Wilks test) and variance (Levene’s test) were evaluated. The SPSS statistic 27 package was used for descriptive statistics and the analysis of variance (three-way ANOVA; environment condition x early life experience x sex). When necessary, Tukey’s Honestly Significant Difference test was used for *post hoc* pairwise comparisons between groups. The results in this section will be expressed as mean ± standard error of the mean, with *p* < 0.05 as statistically significant value. Tables 1, 2 present a summary of the statistical findings.

**TABLE 1:** Summary of 3-way ANOVA for each variable.

Variable	Sex*ELS*EE			Sex*ELS			Sex*EE			ELS*EE		
	DF	F	P	DF	F	P	DF	F	P	DF	F	P
EPM: Percentage of time in open arms	6	0.807	0.565	3	3.287	**0.022**	2	0.215	0.807	6	3.266	**0.004**
EPM: Percentage of open arm entries	6	1.901	0.082	3	3.132	**0.027**	2	3.149	**0.045**	6	1.379	0.224
SI: Interaction index with peer 2	6	0.765	0.598	3	1.049	0.372	2	1.002	0.369	6	0.392	0.884
MWM: Time in the target quadrant	6	2.903	**0.01**	3	0.987	0.4	2	0.808	0.447	6	2.12	0.052
MWM: Cognitive flexibility index	6	0.81	0.564	3	0.736	0.531	2	0.738	0.479	6	3.303	**0.004**
Hippocampal volume	6	0.967	0.454	3	0.573	0.635	2	0.307	0.737	6	1.774	0.117

Note: Summary of 3-way ANOVAs, for parametrical variables. The data is presented by variable (rows) and organized by factor interaction (columns). Abbreviations: DF, degrees of freedom; F the value of Fisher statistics; P for *p*-value, with <0.05 considered as statistically significant. The variables EPM: Percentage of time in open arms and MWM: Cognitive flexibility index were assessed using the transformed variable (sqrt variables).

Bold values represent statistically significant differences.

**TABLE 2:** Summary of Kruskal–Wallis Test for non-parametrical analysis.

Variable	DF	H	P
SI: Interaction index with peer 1	23	44.245	0.005

Note: Summary of Kruskal-Wallis test for non-parametrical variables. The data is presented by variable (rows). Abbreviations DF: degrees of freedom; H; value of H statistics; P for *p*-value, with <0.05 considered as statistically significant.

## 3 Results

### 3.1 Emotional response

#### 3.1.1 Percentage of time spent in the open arms

The anxiety-like behavior was assessed on the EPM test. No statistically significant interaction between the three factors Sex × ELSs × EE was found in the percentage of time spent in the open arms (F [6,209] = 0.807; *p* = 0.565). Significant two-way interactions were found for Sex × ELSs (F [3,209] = 3.287; *p* = 0.022) and EE × ELSs (F [6,209] = 3.266; *p* = 0.004) in the model. Since the three-way interaction was greater than 0.5, we did not remove it from the model and the comparison of marginal means was performed with the full model (Kutner et al., 2005).

**FIGURE 4:**
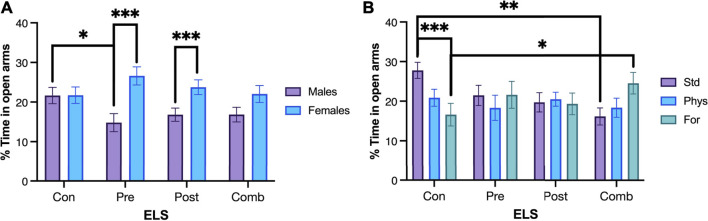
Percentage of time spent in open arms. **(A)** Control males (n = 29) spent more time in the open arms compared with males exposed to prenatal ELS (n = 31). Females exposed to prenatal ELS (n = 28) spent more time in the open arms compared to males exposed to prenatal ELS (n = 31). Females exposed to postnatal ELS (n = 26) spent more time in the open arms compared to males exposed to postnatal ELS (n = 33). **(B)** Control animals spent less time in the open arms when they were housed with Foraging-EE (n = 19) compared to standard housing (n = 19). Rats exposed to combined ELS with standard housing (n = 19) spent less time in the open arms compared to controls (n = 19). Animals exposed to combined ELS and housed with Foraging-EE (n = 18) spent more time in the open arms compared to controls housed with the same Foraging-EE (n = 19). Abbreviations: ELS, Early life stress; Con, Control; Pre, Prenatal; Post, Postnatal; Comb, Combined; EE: Std, Standard; Phys, Physical-EE; For, Foraging-EE. Three Way ANOVA (see Table 1 for statistical results): ***p < 0.001, **p < 0.01, *p < 0.05.

To perform the post hoc methods application and contrast analysis for the model, we compared the estimated marginals means (EMMs) with one another. The Sex × ELSs interaction, showed that the effect of sex depends on the type of ELSs (Figure 4A). Control males spent more time in the open arms compared with males exposed to prenatal ELSs (*p* = 0.009). Additionally, females exposed to prenatal ELSs spent more time in the open arms compared to males exposed to the same condition (*p* < 0.001). A similar pattern was observed for the percentage of time spent in the open arms by females exposed to postnatal ELSs compared to males exposed to the same condition (*p* = 0.01).

Regarding to the EE × ELSs we found that animals that were not exposed to ELSs spent less time in the open arms when they were housed in Physical- EE (*p* = 0.39) and even less time when they were housed in Foraging-EE (*p* < 0.001), compared to the ones with a standard housing as shown in Figure 4B. In the other hand, when the animals were exposed to combined ELSs and housed in Foraging-EE they spent more time in the open arms in comparison with animals with the same ELS and housed in standard condition (*p* = 0 0.024). When the effect of the EE in each level of ELSs was examined, it was observed that, for standard housing (no-EE) the animals exposed to postnatal ELSs showed less percentage of time spent on the open arms (*p* = 0,23) and even less time when they were exposed to combined ELSs (*p* = 0.001) in comparison with animals with no ELSs and housed in the same condition. Additionally, for Foraging-EE the animals exposed to combined ELSs spent more time in the open arms compared to the animals that were not exposed to any ELSs (*p* = 0.009).

### 3.1.2 Percentage of open arm entries

No statistically significant interaction between the three factors Sex × ELSs × EE was found in the percentage of open arm entries (F [6,209] = 1.901; *p* = 0.082). Significant two-way interactions were found for Sex × ELSs (F [3,209] = 3.132; *p* = 0.027) and Sex ×EE (F [2,209] = 3.149; *p* = 0.045) in the model. Although the main factor sex was significant, it was not considered because it interacted with ELS and EE in the model. Since the three-way interaction was less than 0.5, it was eliminated from the model and the comparison of marginal means was performed with a reduced model (Kutner et al., 2005).

**FIGURE 5:**
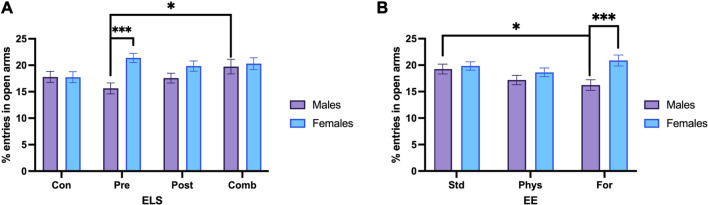
Percentage of open arm entries. **(A)** Males exposed to combined ELS (n = 25) entered more times to the open arm compared to males exposed to prenatal ELS (n = 31). Females exposed to prenatal stress (n = 28) entered more times to the open arm compared to males under the same prenatal stress condition (n = 31). **(B)** Males with standard housing (n = 39) increased the open arm entries, compared to males housed with Foraging-EE (n = 38). Females housed with Foraging-EE (n = 37) showed increased open arm entries compared to males housed with Foraging-EE (n = 38). Abbreviations: ELS, Early life stress; Con, Control; Pre, Prenatal; Post, Postnatal; Comb, Combined; EE: Std, Standard; Phys, Physical-EE; For, Foraging-EE. Three Way ANOVA (see Table 1 for statistical results): ***p < 0.001, *p < 0.05.

To perform the post hoc methods application and contrast analysis for the model, we compared the estimated marginals means (EMMs) with one another. For the Sex × ELSs interaction the analysis showed that females exposed to prenatal ELSs had a significantly increased percentage of open arm entries compared to males (*p* < 0.001). The *post hoc* analysis also showed that males exposed to combined ELSs displayed a significantly increased percentage of open arm entries compared to males exposed to prenatal ELSs (*p* = 0.006; Figure 5A). In the case of females, when they were exposed to prenatal ELS displayed a higher percentage of open arm entries compared to females with no ELS experience (*p* = 0.014).

Regarding to the sex × EE interaction. The *post hoc* comparisons showed that males with standard housing had a significantly higher percentage of open arm entries compared to males that were housed with Foraging-EE (*p* = 0.019). Additionally, females that were housed with Foraging-EE displayed a significantly higher the percentage of open arm entries compared to males housed using the same EE (*p* < 0.001; Figure 5B).

### 3.2 Social response

#### 3.2.1 Interaction index

**FIGURE 6:**
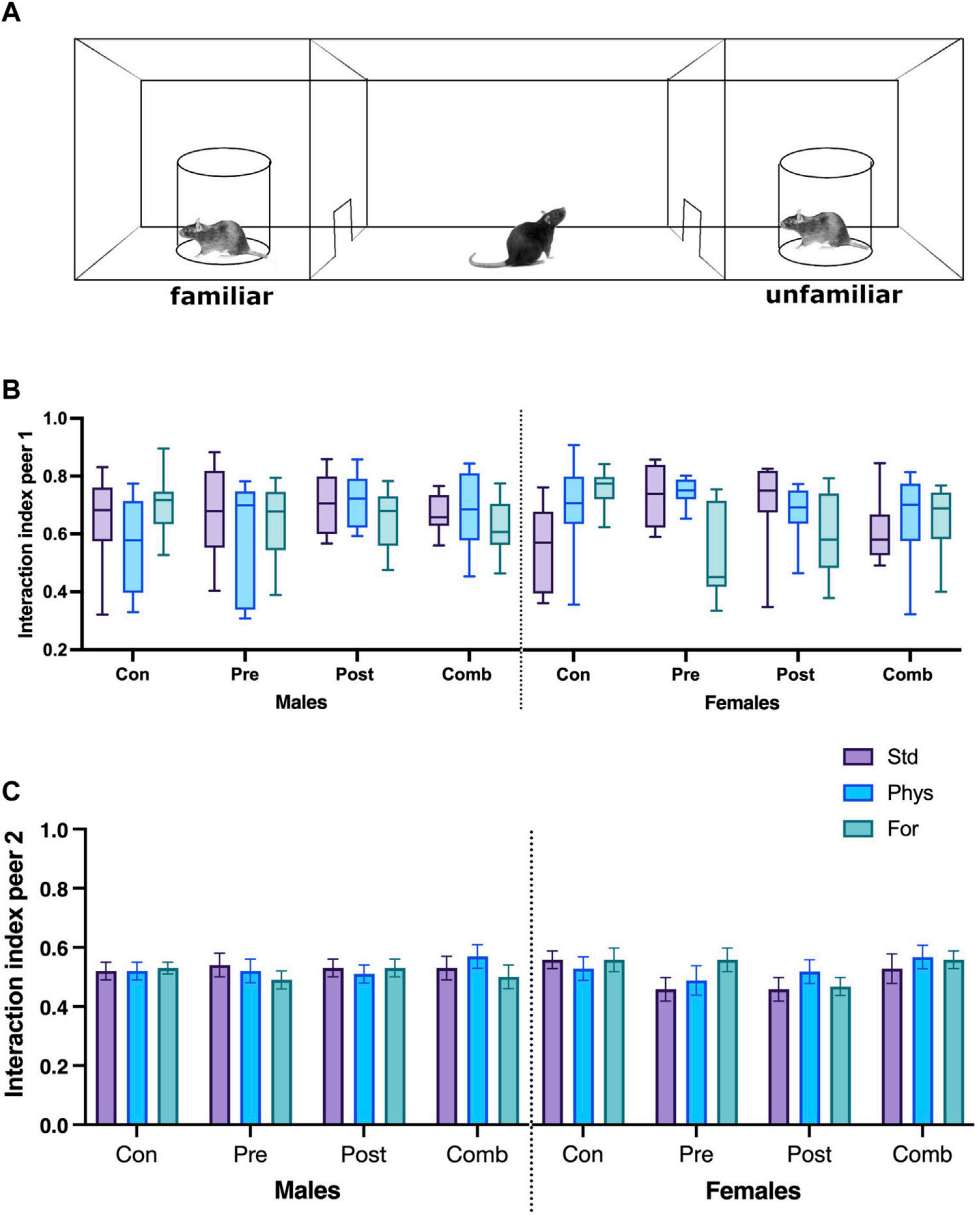
Social interaction test schematic diagram **(A)**. Interaction index with peer 1 **(B)** and peer 2 **(C)**. Abbreviations: ELS, Early life stress; Con, Control; Pre, Prenatal; Post, Postnatal; Comb, Combined; EE: Std, Standard; Phys, Physical-EE; For, Foraging-EE; Comb, Combined. No significant differences were found between the groups (Kruskal–Wallis).

The social behavior was assessed on the SI test. The data analyzed for the interaction index from the first trial did not meet the normality criterion (*p* < 0.001), nor the variance criterion (*p* = 0.003), and the residuals for this variable did not meet the expected normality criterion for ANOVA (*p* = 0.001). Therefore, the non-parametric analysis Kruskal–Wallis was performed, and it showed no significant differences in the comparison of the medians for any group evaluated (Figure 6B).

The interaction index from the second trial (Figure 6C) showed no statistically significant interaction between the three factors Sex × ELSs × EE (F [6,209] = 0.765; *p* = 0.598) or between the two factor interactions.

### 3.3 Memory and cognition

#### 3.3.1 Spatial memory

**FIGURE 7:**
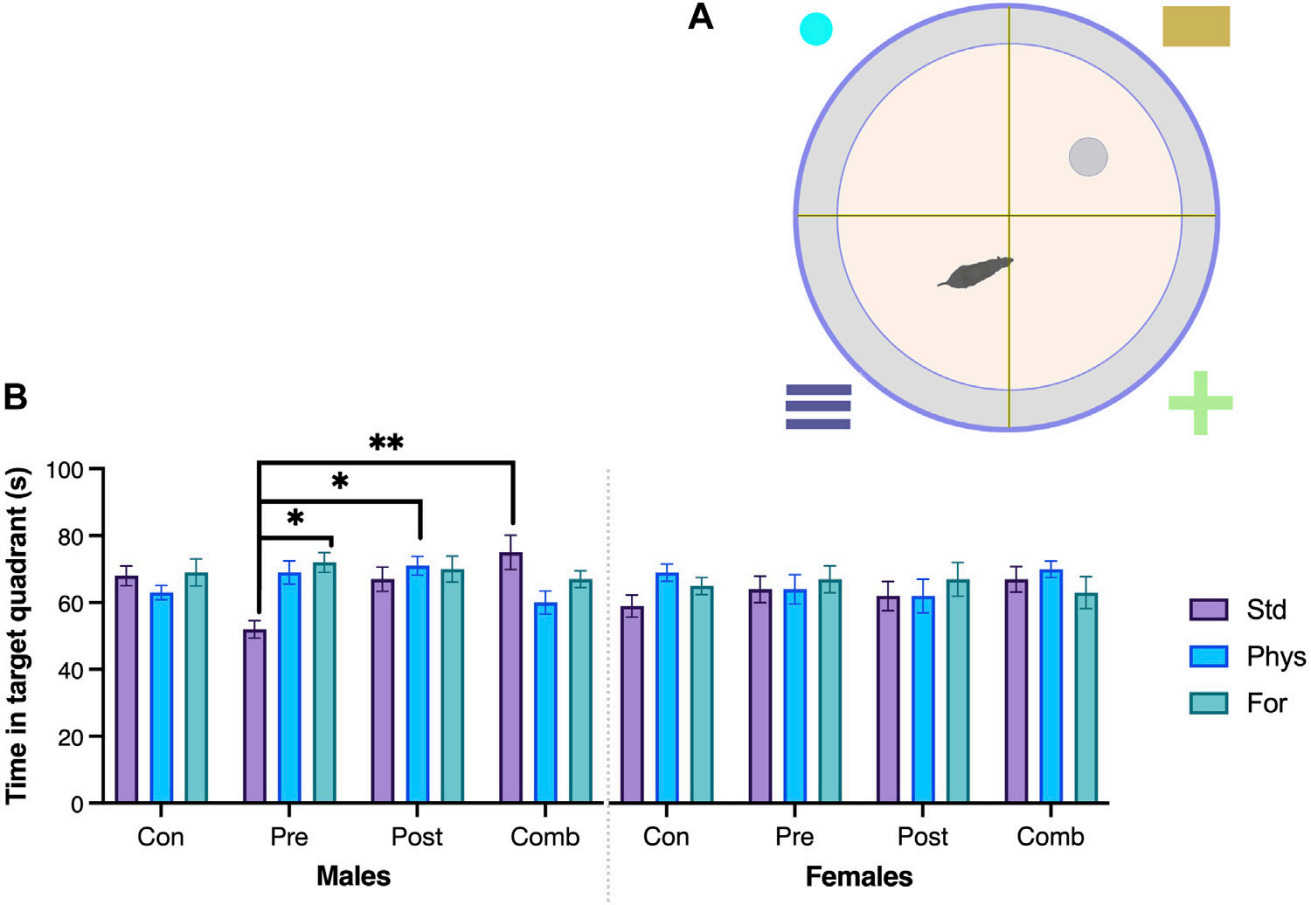
Spatial memory—Morris Water Maze. **(A)** Schematic drawing of the Morris Water maze. **(B)** Time (seconds) in the target quadrant. Males exposed to combined ELS with standard housing (n = 9) spent more time (seconds) in the target quadrant compared to males exposed to prenatal ELS with standard housing (n = 10). Males exposed to prenatal ELS housed with Foraging-EE (n = 8) spent more time (seconds) in the target quadrant compared to males exposed to prenatal ELS with standard housing (n = 10). Males exposed to postnatal ELS housed with Physical-EE (n = 13) spent more time (seconds) in the target quadrant compared to males exposed to prenatal ELS with standard housing. ELS (n = 10). Abbreviations: ELS, Early life stress; Con, Control; Pre, Prenatal; Post, Postnatal; Comb, Combined; EE: Std, Standard; Phys, Physical-EE; For, Foraging-EE. Three Way ANOVA (see Table 1 for statistical results): **p < 0.01, *p < 0.05.

Spatial memory was assessed on the MWM test. The analysis of the time spent in the trained quadrant during the test session (Figure 7) showed that the interaction between the three factors Sex × ELSs × EE was statistically significant (F [6,209] = 2.903, *p* = 0.010). The simple effects test showed that females with no ELS and housed in Physical-EE spent more time in the trained quadrant than females with no ELS housed in standard condition (*p* = 0.034) but when females were exposed to prenatal ELS and were housed in standard conditions spent more time in the trained quadrant compared with males from the same ELS and EE condition (*p* = 0.025).

In case of males exposed to prenatal ELS, when they were housed in standard condition, spent less time in the trained quadrant in comparison with males housed in Physical-EE (*p* < 0.001) and even less time than those housed in Foraging-EE (*p* < 0.001). But when the males were exposed to combined ELS, those housed in standard conditions spent more time in the quadrant compared with males housed in physical-EE (*p* = 0.010). Also, males with history of prenatal ELSs and housed in standard conditions spent less time in the trained quadrant in comparison with males exposed to combined (*p* < 0.001), postnatal ELS (*p* = 0.02) or no ELS at all (*p* = 0.02) when they were also housed in standard. Finally, when males were exposed to postnatal ELS, they spent more time in the trained quadrant in comparison with males exposed to combined ELS when both were housed in Physical-EE (*p* = 0.034).

### 3.3.2 Cognitive flexibility

**FIGURE 8:**
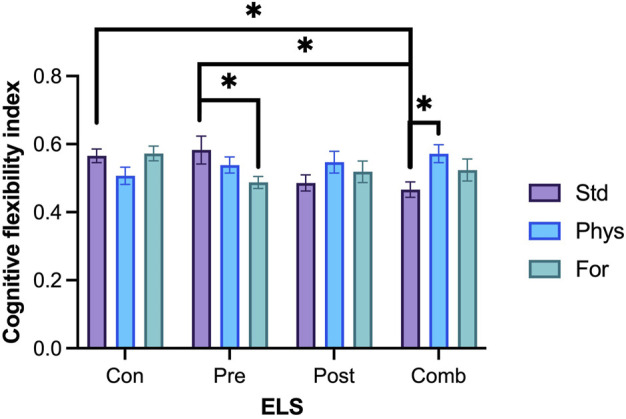
Cognitive flexibility index. Rats exposed to prenatal ELS with Foraging-EE (n = 18) displayed a lower cognitive flexibility index compared to the ones exposed to the same prenatal ELS with standard housing (n = 18). Rats exposed to combined ELS and housed with Physical-EE (n = 16) showed a higher cognitive flexibility index compared to the ones exposed to the same ELS with standard housing (n = 19). Rats exposed to prenatal ELS with standard housing (n = 18) displayed a higher cognitive flexibility index compared to the ones exposed to combined ELS with standard housing (n = 19). Rats exposed to combined ELS with a standard housing (n = 19) showed a lower cognitive flexibility index compared to the ones with no ELS with standard housing (n = 19). Abbreviations: ELS, Early life stress; Con, Control; Pre, Prenatal; Post, Postnatal; Comb, Combined; EE: Std, Standard; Phys, Physical-EE; For, Foraging-EE; Comb, Combined. Three Way ANOVA (see Table 1 for statistical results): *p < 0.05.

Cognitive flexibility was assessed on the MWM test, using a cognitive flexibility index (average arrival latencies to the platform on the last training session and the first reversal session, Figure 8). No statistically significant interaction between the three factors Sex × ELSs × EE was found (F [6,209] = 0.81; *p* = 0.564). Significant two-way interactions were found for EE × ELSs (F [6,209] = 3.303, *p* = 0.004). Since the three-way interaction was close to 0.5, we did not remove it from the model and the comparison of marginal means was performed with the full model (Kutner et al., 2005). To perform the post hoc methods application and contrast analysis for the model, we compared the estimated marginals means (EMMs) with one another. Regarding to the housing condition, the analysis revealed that animals housed in standard conditions and exposed to combined ELSs showed a lower cognitive flexibility index compared to animals with prenatal ELS (*p* = 0.113) and with no ELSs (*p* = 0.100) in standard housing. In the same line, animals exposed to postnatal ELS and housed in standard conditions showed a lower cognitive flexibility index compared to those with prenatal ELS (*p* = 0.095) o no ELS at all (*p* = 0.082). Finally, animals with no ELS showed a higher cognitive flexibility index than those exposed to prenatal ELS when they are housed in foraging-EE conditions (*p* = 0.084).

When we explore for each level of ELS, we found that animals exposed to combined ELSs and housed with Physical-EE had a higher cognitive flexibility index compared to the ones exposed to the same ELSs but with a standard housing (*p* = 0.109). But when animals were exposed to prenatal ELSs and housed in standard condition they had a higher cognitive flexibility index compared to the ones exposed to the same ELSs and housed in Foraging-EE (*p* = 0.093).

### 3.4 Neuroanatomical changes

#### 3.4.1 *Hippocampus* volume

**FIGURE 9:**
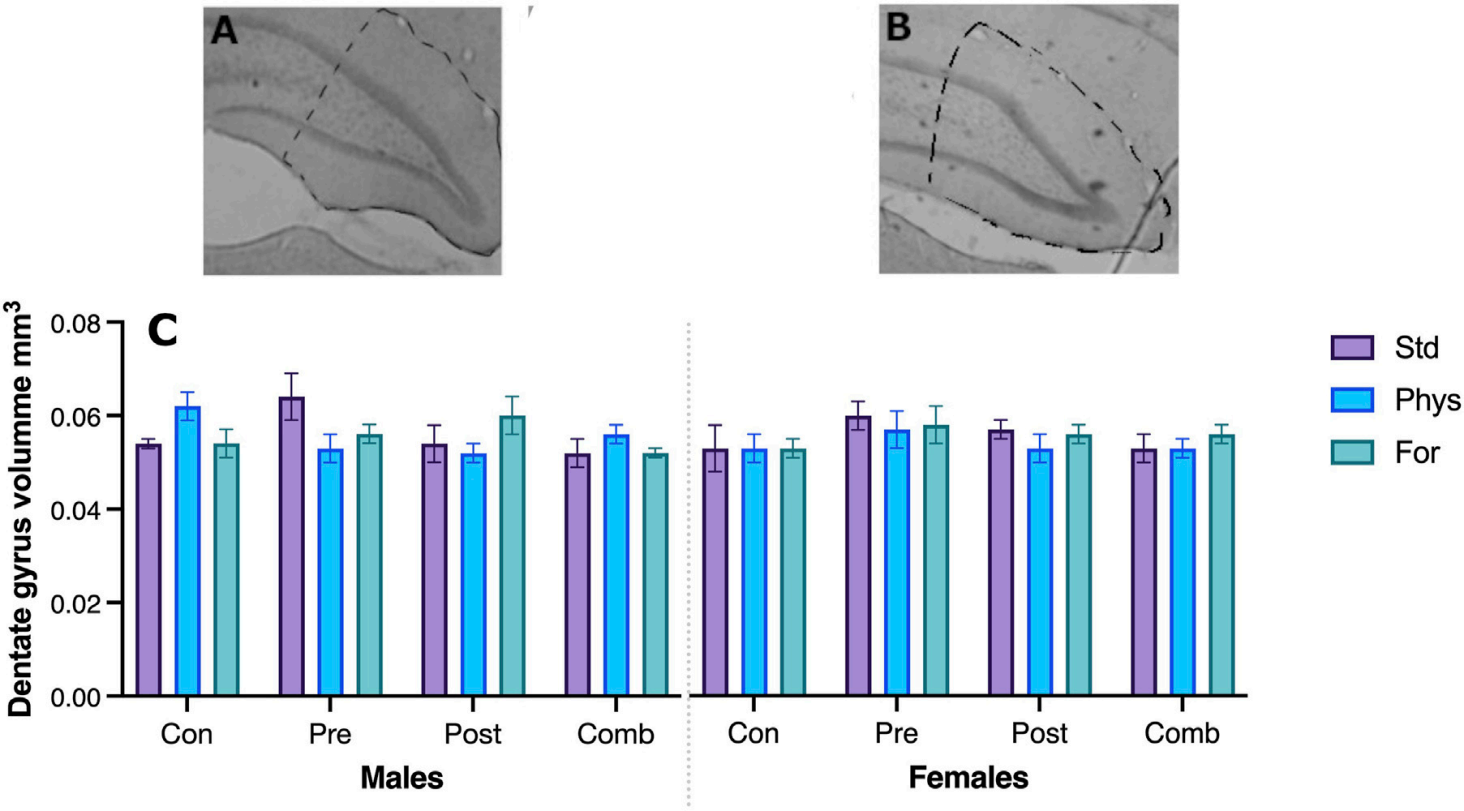
*Hippocampus* dentate gyrus in a typical control **(A)** and ELS **(B)** subject. The volume of the hippocampus was not statistically different between the groups **(C)**.

No significant interaction between the three factors Sex × ELSs × EE was found in the volume of the dentate gyrus (F [6,69] = 0.967; *p* = 0.454). The interactions between Sex × ELSs (F [3,209] = 0.573, *p* = 0.635), EE × ELSs (F [6,209] = 1.774, *p* = 0.117), and Sex × EE (F [2,209] = 0.307, *p* = 0.737) were not statistically significant. See Figure 9 for a picture of typical dentate gyri in control and ELS animals.

## 4 Discussion

This work analyzed the effect of three types of adverse early-life experiences and two types of environmental enrichment on emotional, social, and cognitive responses of male and female Wistar rats. A significant effort was made in terms of the control of variables, including the restriction of staff who had contact with the animals, as well as to permanently guarantee the health and welfare of the animals.

The complexity of the stressors and their effects in terms of magnitude, time bin, implementation strategy, and dimension evaluated cover a spectrum that is difficult to consider when formulating an experimental design. Here we chose to use stimuli whose intensity could be precisely controlled, seeking to simulate conditions normally encountered in human experiences. This selection of the ELS protocol allowed us to arrive to a situation closer to the real human conditions.

During our entire experiment, permanent monitoring of the pregnant animals and the offspring was carried out. The establishment of safe parameters for the implementation of prenatal and lactational stress protocols led us to choose moderate stressors that did not compromise the well-being and health of the animals in the short and long term.

Some studies use “*severe*” stress inducers, such as durations longer than 3 h per day of maternal stress through physical immobility. Others use important alterations in the social environment of the pregnant animal such as change of companions, and situations of social intrusion, yet others involve the daily administration of electric shocks during important periods of gestation. Such strong stressors can compromise the integrity of the fetuses and even result in a termination of the gestational process. In our work, the behavior of the pregnant females was carefully observed before and during the implementation of the stress protocol, and the females were weighed daily to detect abrupt weight loss. The period of physical immobility was distributed in three moments of the day (45 min each), during the active phase of the animals. In this way, the access of the pregnant female to food and water was guaranteed, without the physical restriction having important effects on the development of the fetuses. Despite these considerations, there was loss of offspring during gestation and lactation. However, this loss of neonate animals was as low as in the normal breeding condition (4%).

Regarding postnatal stress, the strategy used here was maternal separation. This type of early stress involves depriving the offspring of maternal care for several hours per day, during the early and middle phases of the lactation period. Studies implementing maternal separation stress can go as far as using periods of up to 6 h over several days, or a single extended period of deprivation of up to 24 h at a time. Such approaches do not consider the nutritional, toxic, and thermal effect this procedure may have on the offspring, so the trade-off, in terms of animal welfare, would be questionable in these models. In presence of more severe stressors, the effects of the stress protocols will be much easier to identify since the disruption in the welfare of the animals and the strategies that they will deploy to compensate will be more explicit at physiological and behavioral levels. As already mentioned, this work was aimed to the simulation of a situation as close as possible to human conditions, without interfering with the anatomical and physiological integrity of the animals.

Regarding the environmental enrichment conditions used, as already stated, one of the reasons for the enormous variation of effects of environmental modifications on behavior found in the literature, is the lack of systematic efforts to properly analyze the contribution of the various features of stimuli used as enrichers. Looking for a more detailed comprehension of the effect of different enrichers on behavior here we used two kinds of enrichers: one of them eliciting active behavior (active enrichment: foraging) while the other did not elicit active behavior (passive enrichment: physical).

### 4.1 Emotional responses: Anxiety

Our data confirmed a positive effect of environmental enrichment on anxiety-like behaviors in animals with ELSs. This effect depends on the type of experience and enrichment that the animal was submitted to. We found lesser anxiety-like behaviors in the EPM in animals with prenatal and postnatal stress and housed in environments with presence of foraging enrichment. Interestingly, foraging-type enrichment can also induce a greater anxiety-like response in animals without adverse experiences (Figures 4, 5). The effects of foraging enrichment are also sex dependent: females showed lower anxiety-like behaviors compared to males, while males housed in foraging EE showed higher anxiety-like behaviors compared to males housed in standard conditions.

It is worth keeping in mind that a decrease in the percentage of entries to open arms (Figure 5) has been associated with an increase in anxiety levels (Campos et al., 2013; Bourin, 2015; de Sousa et al., 2015) and/or a decrease in exploratory interest (Cardenas et al., 2001; Lamprea et al., 2010; Tejada et al., 2010). Males housed in the foraging condition are likely to have a decreased interest in exploring the maze because the very housing conditions provided enough exploration opportunities to solve the natural conflict between exploration and anxiety presented in the elevated plus maze. Unfortunately, there are no reports in the literature about this relationship, due to of the absence of use of foraging as an environmental enrichment strategy. Our results may help to shed some light in understanding this relationship.

The differential effect of foraging EE could depend on behavioral needs of males and females. In the case of males, the presence of foraging may have created competitive relationships between them, working as an element that alters social organization diminishing the interest in exploration or enhancing their anxiety-like behavior. In the case of females, the foraging material could possibly be seen as an improvement in resource availability and as opportunities for interaction through the shared use of resources (i.e., in nature, nests are usually communal). In any case, the lack of literature on the use of foraging as EE, makes it hard to determine a direct explanation for this effect.

Here we propose that an active EE, with which the animal interacts, could reduce the need for exploration providing a sense of control of the environment and originating new interaction possibilities, even problem-solving strategies. Supporting the idea of a differential effect on males and females on exploration, our results suggest a stronger effect in females, i.e., for all types of stress, females showed an increase in the percentage of entries to open arms when they received foraging enrichment.

Another aspect worth mentioning, is the interactions between sex and ELSs. Our findings suggest a sex diverging effect: a cumulative anxiolytic effect for males, and a non-significant trend to a cumulative anxiogenic-like effect for females. This could possibly indicate lower vulnerability in males for the two combined ELSs. There are some reports on sexual differential vulnerability on the effects of prenatal and postnatal stress on anxiety responses (Lam et al., 2018, 2019; Soares-Cunha et al., 2018; Bassey and Gondre-Lewis, 2019; Liao et al., 2019; Huerta-Cervantes et al., 2020). It is also possible that the combined ELSs cause divergent effects only on some tests, but a conclusive explanation for this phenomenon has not been achieved (Sparling et al., 2018; Bassey and Gondre-Lewis, 2019).

Our finding of a decreased anxious response in females submitted to pre- and postnatal stress confirms previous reports regarding performance in the elevated plus maze, this in terms of higher levels of exploration by females compared to males, even after early stress experiences (Scholl et al., 2019). According to previous evidence, the exploratory pattern of females could be an activity-related behavior to foraging for food resources or nest building, and could be enhanced when rats are subjected to deprivation of these resources early in life (Prusator and Greenwood-Van Meerveld, 2015; Maniam et al., 2016).

As previously mentioned, the prenatal stress protocol used here is can be considered as a “*mild*” protocol when compared to other protocols, which can be considered as “*severe*”, including prenatal exposure to drugs, nicotine or ethanol, induction of diabetes, for example (Parks et al., 2008; Schroeder et al., 2013; Galea et al., 2014; Ratajczak et al., 2014; An and Zhang, 2015). For this reason, it can be proposed that its effect on anxiety is not very strong - hence leading to non-significant differences when comparing stressed vs non-stressed groups - but sufficiently pronounced to show significant differences when comparing to the group of females in the same conditions.

### 4.2 Social responses: Social interaction

Our results suggest that social behavior is independent of both the ELSs and the EE factors assessed in this work. The study of social behavior is very challenging; its expression varies depending on many factors, including the test used, the characteristics of the subjects used as pairs (familiar or strangers, strain, place in the social hierarchy, stress history, age, and sex), and the complexity of the environments where the test animals are housed, among other elements (Beery and Kaufer, 2015).

The social interaction test proposed by Crawley was initially considered as a strategy to evaluate a mouse model of autism. It aims to quantify peer-directed behaviors, exploration of odor cues, ultrasonic vocalizations, and stereotypies (Crawley, 2007). Here, this test was adapted to evaluate rats, quantifying two features of social responses: interest and social discrimination (Kentrop et al., 2018; Kambali et al., 2019). It is noteworthy, that positive social behaviors were comparable in males and females evaluated in each stress and environmental enrichment condition.

As pointed by some authors, early stress events alter the social performance of subjects during adulthood (Holland et al., 2014; Kompier et al., 2019). However, the relationship in terms of the magnitude of the stressor and the magnitude of the response is something that has not been previously addressed. Many early stress protocols use periods of deprivation or presence of the stressor far superior to what might occur in nature (Kentrop et al., 2018; Kambali et al., 2019), affecting the analyzed responses and limiting the translational potential of the data. On the other hand, the increase in maternal care behaviors after prolonged periods of separation may reduce the effect of a severe stressor on adults’ social responses (Beery and Kaufer, 2015), leading to increased social interaction.

The absence of changes in social interactions indicates that neither physical nor foraging EE protocols used here could affect the genesis of social behavior. This is a very relevant finding because it has been previously reported that some environmental enrichment protocols (using a diversity of stimuli) could affect social performance, depending on factors such as the time window of exposure and the social dimension tested (Mesa-Gresa et al., 2013; Connors et al., 2015).

We can conclude that social responses assessed by the test in this work, did not change because of the mild quality of the stress protocol used. Moreover, the stability of social responses among the groups allows us to conclude that at least the types of enrichments (physical and foraging) and the three types of stress (prenatal, postnatal, and combined) used here have no direct effect on the social responses of animals. New studies on the effect of stronger ELSs protocols and of different controlled categories of environmental enrichment stimuli on this social test must be conducted to clarify this relationship.

### 4.3 Cognitive responses

#### 4.3.1 Spatial memory

In line with the proposed hypothesis, it was observed that environmental enrichment has a positive effect on the spatial memory of subjects (Figure 7). The effect of foraging EE is positive for males with a history of prenatal stress, and physical EE is positive those for males with a history of postnatal stress.

The time spent in the target quadrant, during the MWM test has been used in the assessment of hippocampal function in rats (Weinstock, 2017; Benmhammed et al., 2019). Many reports describe the effect of early stressors on this response (Couto et al., 2012; Diehl et al., 2012). These studies usually use severe stressors, such as anticonvulsant drugs (Ishola et al., 2020), amphetamine (Holubová et al., 2018), ultrasound exposure (Abramova et al., 2020), maternally induced diabetes (Huerta-Cervantes et al., 2020), gamma exposure (Pipová Kokošová et al., 2020), or chronic changes in maternal diet during gestation (Bengoetxea et al., 2017). Such procedures cause serious cellular alterations in many brain regions, including the hippocampus, and the results found in the MWM may reflect these structural alterations. The effect of EE on the recovery of conditions related to ischemia, chronic stress, and mild cognitive impairment (Dahlqvist et al., 2004; Hullinger et al., 2015; Bhagya et al., 2017), as well as adverse early life experiences (Hui et al., 2011; Vivinetto et al., 2013; do Prado et al., 2016), has also been describe in the literature.

Here we observed the positive effects of environmental enrichment on spatial memory performance in animals who had experienced stress. Foraging EE was positive for recovering the effect of prenatal stress in males, while physical enrichment was positive for males with a history of postnatal stress.

The use of EE as an intervention strategy seeks to reverse or mitigate the negative effects of adverse events. In the case of postnatal stress, enrichment has been documented to successfully reverse or mitigate the effects of maternal separation or parenting with limited resources (Cui et al., 2006; Dandi et al., 2018). Also, there are many reports on environmental enrichment showing that it has positive effects on the recovery of functions associated with spatial memory in animals exposed to prenatal stress (Zhang et al., 2012; Nowakowska et al., 2014).

The use of moderate stressors, such as maternal restraint for periods of 3 h per day, causes some changes in aspects such as the latency of arrival at the MWM platform or the amount of crossover due to its previous location (Chen et al., 2017; Su et al., 2019). However, many studies have found that this type of prenatal stress does not cause changes in the time spent by animals in the trained quadrant in the test session (Chen et al., 2017; Weinstock, 2017). In line with these reports, our results confirm the “moderate” quality of the maternal separation protocol that was used.

In the literature, there are very few reports on the differential effect of prenatal maternal restraint stress on males and females (Zuena et al., 2008). Although, there is some consensus about the effect being more negative in younger animals, even more in younger females (Zuena et al., 2008; Weinstock, 2011), this effect was not possible to identify in our work because age was not used as a an experimental variable and for that reason all animas were trained and tested in the MWM with exactly the same age.

It has also been described that the effects of prenatal stress on memory are strongest between 3 and 8 weeks, after that the effect begins to fade (Yaka and Weinstock, 2007; Barzegar et al., 2015). Due to our experimental design, it was required that the animals were initially evaluated in the elevated plus maze (as it is a test very sensitive to other manipulations), then in the social interaction test, and finally in the MWM. At the time of the performance of the MWM platform test, the animals were 8 weeks old; thus, it is possible that at this point the effect of the stress could have been fading.

#### 4.3.2 Cognitive flexibility

Our data confirm a positive effect of environmental enrichment on cognitive flexibility in animals subjected to ELSs. Such effect depends on the type of stress experienced, and the type of enrichment implemented (Figure 8). Animals with a history of combined stress and exposed to physical enrichment showed a higher index of cognitive flexibility than their standard housed counterparts. This could indicate that physical enrichment is able to mitigate the effect of combined stress on the cognitive flexibility response.

However, when animals were housed in standard conditions (without environmental enrichment), those exposed to combined stress had lower cognitive flexibility index in comparison to animals exposed to prenatal stress and animals with no stress experience. This may indicate that combined stress without any environmental enrichment is more adverse than prenatal stress or control rearing.

Our results are in line with evidence from other researchers. For instance, in the work of Menezes et al. a shorter maternal separation protocol (PND 1–10) with the same daily duration as in our work was evaluated. Their findings confirm a protective effect of enrichment on cognitive flexibility assessed in the MWM (Menezes et al., 2020). These effects have also been documented in studies including perinatal exposure to toxic substances, perinatal oxygen deprivation, maternal deprivation, among others (Baudin et al., 2012; Galeano et al., 2014; Sampedro-Piquero et al., 2015; Aung et al., 2016; Thomas et al., 2016; Kambali et al., 2019) in which EE manage to recover the cognitive flexibility response in behavioral paradigms such as the MWM (Halperin and Healey, 2011; Redolat and Mesa-Gresa, 2011; Saland and Rodefer, 2011). However, studies involving the effect of stressors such as physical restraint of the pregnant mother or maternal separation on cognitive flexibility are rare. In fact, an article database search that includes prenatal stress, cognitive flexibility, and environmental enrichment as keywords showed no results. For this reason, our data could serve as a starting point for the study of this relationship.

### 4.4 Hippocampus

The absence of structural changes is consistent with the data in relation to spatial memory, supporting the idea that the level of stress induced corresponds to moderate. Indeed, moderate stress has not been reported to be associated with anatomical alterations of the hippocampus, in contrast to severe cases of early stress.

The first studies that looked for anatomical effects of environmental enrichment in rats date back to 1976. Diamond and coworkers exposed rats to enriched and impoverished environments for prolonged periods and found that exposure to the impoverished environment caused a decrease in the thickness of the cerebral cortex. Their studies showed no evidence that exposure to enriched environments caused changes in the hippocampus, although improvements in memory were found (Diamond et al., 1972). Years later it was reported that changes were only present in the hippocampus of subjects from the impoverished environment (isolation), and these changes might be more related to the size of the cell nuclei (Walsh and Cummins, 1979). From these initial works, attempts have been made to establish causal relationships between environmental changes and the hippocampus, given the great evidence environmental enrichment effects on memory (Barros et al., 2019).

Our data did not show anatomical changes in the volume of the dentate gyrus associated with any of the factors. This does not exclude the possibility of other changes at a finer structural level. To determine whether the adverse experiences used here were of great severity, we opted for this global measure, since it has been reported that early exposure to strong traumatic events causes a decrease in hippocampal volume, both in humans and in rodents (Sheline, 1996; Gorka et al., 2014; Schoenfeld et al., 2017). Further studies at a finer level (i.e., cellular, and molecular) must be conducted.

## 5 Conclusion

Our results confirmed that the prenatal and postnatal stress protocol used here correspond to a non-severe type of stress. As a result, we did not find huge changes in anatomy—inducing alterations in the volume of the dentate gyrus—like those reported after the use of severe conditions such as exposure to anticonvulsants, and addictive drugs (i.e., amphetamine), ultrasounds, or gamma radiation exposure.

The key element of our experiment was the use of environmental enrichment as an intervention to recover from the adverse effects of early mild experiences. It is clear from the literature review that the stimuli commonly used as enrichers are not targeting any precise function. For example, the use of tactile, auditory (including music), and visual stimuli (including color variation) has been documented in Wistar strain rats - known for their limited visual capacity - and without consideration of the audible frequency ranges or in general the correspondence between the sensory features of the stimuli and the perceptual capabilities of the animals. These practices reduce the comparability of the data and limit the possibility of generalizing the effects across research. In addition, it is difficult to find studies that use foraging-type enrichment, so our work underscores the need to use of function-specific protocols of environmental enrichment. Of course, more studies discriminating different kinds of function-specific enrichers need to be conducted. Nevertheless, our proposal of function-specific enrichments may help to improve this aspect of research.

The environmental enrichment strategies proposed here aim to be as specific as possible in terms of function and form, providing the animals with elements designed for specific purposes, ensuring novelty throughout the treatment. Our enrichment devices separately aimed to target the structural modification of the environment by adding PVC material of different sizes and shapes and at the availability of materials for nest construction. Each of these elements was selected considering the safety and creation of satisfactory environments in terms of access to resources, social needs, and space. The physical enrichment work as a ***passive enrichment*** since it cannot be manipulated by the subject and modifies the architecture of the subjects’ accommodation. Foraging type enrichment, on the other hand, served as an ***active enrichment***, since it can be manipulated by the subject without modifying the architecture of the home cage. The choice of these two categories of environmental enrichment were based on previous research conducted at our laboratory. The selection of the enrichers was carefully made considering the characteristics of the species, the nature of the responses evaluated, and the way these are shaped in natural environments. Aspects such as the age of the subjects, the period of exposure, and the experimental history of the subjects were also considered.

Our data confirm that, it is feasible to work with females in behavioral studies and obtain conclusions that can contribute to the body of knowledge of the discipline, making a clear distinction between the effects of independent variables between males and females. It is necessary to continue promoting the creation of research designs including the sex variable, since the goal of biomedical research is human welfare, and this includes both men and women. We hope that this work will contribute to the construction of a more complete picture of the environment effects on behavior.

## Data Availability

The raw data supporting the conclusions of this article will be made available by the authors, without undue reservation.
